# KIR3DL05 and KIR3DS02 Recognition of a Nonclassical MHC Class I Molecule in the Rhesus Macaque Implicated in Pregnancy Success

**DOI:** 10.3389/fimmu.2022.841136

**Published:** 2022-03-23

**Authors:** Rachel E. Nicholas, Kjell Sandstrom, Jennifer L. Anderson, Willow R. Smith, Molly Wetzel, Priyankana Banerjee, Sanath Kumar Janaka, David T. Evans

**Affiliations:** ^1^Department of Pathology and Laboratory Medicine, University of Wisconsin-Madison, Madison, WI, United States; ^2^Wisconsin National Primate Research Center, University of Wisconsin-Madison, Madison, WI, United States

**Keywords:** KIR, MHC, Mamu-AG, macaque, HLA-G, placenta, pregnancy

## Abstract

Knowledge of the MHC class I ligands of rhesus macaque killer-cell Ig-like receptors (KIRs) is fundamental to understanding the role of natural killer (NK) cells in this species as a nonhuman primate model for infectious diseases, transplantation and reproductive biology. We previously identified Mamu-AG as a ligand for KIR3DL05. Mamu-AG is a nonclassical MHC class I molecule that is expressed at the maternal-fetal interface of the placenta in rhesus macaques similar to HLA-G in humans. Although Mamu-AG and HLA-G share similar molecular features, including limited polymorphism and a short cytoplasmic tail, Mamu-AG is considerably more polymorphic. To determine which allotypes of Mamu-AG serve as ligands for KIR3DL05, we tested reporter cell lines expressing five different alleles of KIR3DL05 (KIR3DL05*001, KIR3DL05*004, KIR3DL05*005, KIR3DL05*008 and KIR3DL05*X) for responses to target cells expressing eight different alleles of Mamu-AG. All five allotypes of KIR3DL05 responded to Mamu-AG2*01:01, two exhibited dominant responses to Mamu-AG1*05:01, and three had low but detectable responses to Mamu-AG3*03:01, -AG3*03:02, -AG3*03:03 and -AG3*03:04. Since KIR3DL05*X is the product of recombination between *KIR3DL05* and *KIR3DS02*, we also tested an allotype of KIR3DS02 (KIR3DS02*004) and found that this activating KIR also recognizes Mamu-AG2*01:01. Additional analysis of Mamu-AG variants with single amino acid substitutions identified residues in the α1-domain essential for recognition by KIR3DL05. These results reveal variation in KIR3DL05 and KIR3DS02 responses to Mamu-AG and define Mamu-AG polymorphisms that differentially affect KIR recognition.

## Introduction

MHC class I molecules play a central role in both innate and adaptive immunity. In addition to presenting peptides derived from intracellular antigens on the cell surface for recognition by CD8^+^ T cells, MHC class I molecules serve as ligands for killer cell Ig-like receptors (KIRs) on natural killer (NK) cells (1, 2). Whereas the extensive polymorphism of classical MHC class I molecules ensures the presentation of a diverse repertoire of peptides derived from intracellular pathogens to CD8^+^ T cells, the limited polymorphism of nonclassical MHC class I molecules reflects their more specialized functions.

Macaques and other Old World monkeys express an expanded array of polymorphic *MHC class I* genes that correspond to the classical *HLA-A* and *-B* genes of humans (3–5). However, these species lack an ortholog of *HLA-C* as a consequence of the recent evolutionary origin of *HLA-C* as a duplication of an ancestral *MHC-B* gene that occurred after the divergence of apes and Old World monkeys (3, 6). Old World monkeys have accordingly expanded their lineage II *KIR*, which encode KIR3D receptors for MHC-A and -B, but lack lineage III *KIR* that encode KIR2D receptors for HLA-C in humans (7–13). Old World monkeys also have orthologs of each of the human nonclassical *HLA-E, -F* and *-G* genes. *MHC-E* and *-F* are well-conserved and broadly expressed in primates (14–16). However, the *MHC-G* genes of Old World monkeys have accumulated multiple stop codons and no longer encode functional proteins (17, 18). In the absence of a functional *MHC-G* locus, Old World monkeys have evolved another nonclassical MHC class I gene, designated *MHC-AG*, which appears to serve a similar function (19–22).

Macaques, vervets, and baboons express MHC-AG molecules with similar molecular features and tissue distribution as HLA-G (22, 23). *MHC-AG* sequences are more similar to *MHC-A* than to *MHC-G* as a consequence of their evolutionary origins as an *MHC-A* gene duplication (19–22). However like HLA-G, MHC-AG exhibits limited polymorphism, a truncated cytoplasmic tail, and is only expressed on placental trophoblasts that form the barrier between the mother and the developing fetus (19, 20, 23). Hence, Mamu-AG is believed to serve a similar function as HLA-G, namely contributing to pregnancy success through interactions with NK cells and myeloid cells that promote placental vascularization, tolerance to the haploidentical fetus, and protect the maternal-fetal interface from invading pathogens (23, 24).

The rhesus macaque (*Macaca mulatta*) is an important animal model for infectious diseases such as HIV/AIDS, CMV, Zika virus and SARS-CoV-2 (25–35), transplantation (36, 37), and reproductive biology (23, 38). We previously identified Mamu-AG as a ligand for KIR3DL05 (39). Here we show that KIR3DL05 recognizes certain allotypes of Mamu-AG, but not others, and define residues in the α1- and α2-domains of Mamu-AG essential for interactions with this receptor. We further identify Mamu-AG as a ligand for the activating receptor KIR3DS02. These observations provide an essential foundation for investigating the role of NK cells in the rhesus macaque as a nonhuman primate model for infectious diseases and reproductive biology. These findings also illustrate how polymorphisms in the α1-domain can lead to variation in KIR recognition of closely related MHC class I ligands.

## Materials and Methods

### MHC Class I-Transduced 721.221 Cells

Codon-optimized cDNA sequences encoding Mamu-AG molecules were synthesized by GenScript. These sequences were then cloned into the retroviral vector pQCXIP. Retroviral vectors were packaged into VSV-G-pseudotyped murine leukemia virus (MLV) particles by co-transfecting GP2-293 cells with pQCXIP-Mamu-AG constructs and pVSV-G (Clontech Laboratories). The cell culture supernatant was collected two days after transfection and cellular debris was removed by centrifugation followed by filtration through a 0.45 µm nylon membrane. HLA-null 721.221 cells were transduced by spinoculation for one hour with supernatant collected from the transfected GP2-293 cells. After two days, the transduced cells were placed under antibiotic selection with RPMI medium supplemented with 10% FBS, L-glutamine, penicillin, streptomycin (R10) and 0.4 µg/ml puromycin (Calbiochem). The concentration of puromycin in the medium was gradually increased to 1.0 µg/ml over 3-4 weeks to eliminate untransduced parental 721.221 cells. After 3-4 weeks of selection, cell lines were maintained in medium containing 0.4 µg/ml puromycin. Mamu-AG expression was confirmed by flow cytometry *via* surface staining with a PE-conjugated, pan-MHC class I-reactive monoclonal antibody (W6/32, Life Technologies). Mamu-AG-transduced 721.221 cell lines were maintained in R10 medium containing 0.4 µg/ml puromycin. The GenBank accession numbers for *Mamu-AG* alleles are as follows: Mamu-AG2*01:01 (U84783.1), -AG3*02:01:01:01 (U84785.1), -AG3*02:02:01 (U84786.1), -AG3*03:01:01:01 (U84787.1), -AG3*03:02 (U84789.1), -AG3*03:03 (KF855159.1), -AG3*03:04 (KF855160.1), and -AG1*05:01 (FJ409466). Only partial-length cDNA sequences were available for Mamu-AG3*03:03, -AG3*03:04, and -AG1*05:01. For constructs expressing these alleles, sequences encoding the α3-, transmembrane and cytoplasmic domains were therefore inferred from the *Mamu-AG* consensus sequence.

### KIR-CD3ζ-Transduced Jurkat NFAT Luciferase Reporter Cells

Constructs for the expression of chimeric KIR-CD3ζ receptors with an N-terminal Flag-tag were engineered by cloning cDNA sequences into pQCXIP encoding the D0, D1, D2 and stem domains of KIR3DL05 downstream of sequences for the KIR3DL05*008 leader peptide and Flag-tag (DYKDDDDK) and upstream of sequences coding for the transmembrane and cytoplasmic domains of CD3ζ. Retroviral vectors were packaged into VSV-G-pseudotyped MLV particles as described above. Jurkat NFAT luciferase (JNL) cells (Signosis) were transduced by spinoculation for one hour with filtered supernatant from transfected GP2-293 cells. Two days after transduction, the JNL cells were placed under antibiotic selection in R10 medium containing 100 µg/ml hygromycin to maintain the luciferase reporter gene and 0.5 µg/ml puromycin to select for KIR-CD3ζ expression. The puromycin concentration in the medium was gradually increased to 1.0 µg/ml over 3-4 weeks to eliminate untransduced cells and then maintained at a concentration of 0.4 µg/ml. GenBank accession numbers for rhesus macaque KIR sequences are as follows: KIR3DL05*001/KIR3DLW36*001 (EU419045), KIR3DL05*004 (EU419066.1), KIR3DL05*005/KIR3DLW37*001 (EU419069), KIR3DL05*X (EU419067.1), KIR3DL05*008 (GU112291), and KIR3DS02*004 (EU419026).

### Ligand Identification Assay

KIR-CD3ζ^+^ JNL cells (1x10^5^) were co-cultured with Mamu-AG^+^ or parental 721.221 cells (1x10^5^) overnight at 37°C and 5% CO_2_ in triplicate wells of white 96-well plates in 100 µl R10 medium without antibiotics. As a positive control, KIR-CD3ζ^+^ JNL cells were treated with 5 µg/ml of an anti-Flag-tag monoclonal antibody (GenScript) and 10 µg/ml of a goat anti-mouse secondary antibody (Poly4053, Biolegend). After 12-18 hours, 100 µl of BriteLite Plus luciferase substrate (PerkinElmer) was added to each well and luciferase activity in relative light units (RLU) was measured using a VICTOR X4 multiplate reader (PerkinElmer).

### Flow Cytometry

KIR-CD3ζ expression was verified by surface staining parental and KIR-CD3ζ-transduced JNL cells with a PE-conjugated anti-Flag antibody (Miltenyi Biotec). MHC class I expression was confirmed by surface staining parental and Mamu-AG-transduced 721.221 cells with a PE-conjugated pan-MHC class I-specific monoclonal antibody (W6/32, eBioscience). JNL and 721.221 cells were also stained with live-dead near-IR fluorescent stain (Invitrogen) to exclude dead cells. Data was collected on a BD FACSymphony flow cytometer and analyzed using FlowJo software (TreeStar, Inc.). After gating on singlet, viable cells, the surface expression of KIR-CD3ζ and Mamu-AG on transduced cells was compared to parental JNL and 721.221 cells.

### Statistical Analysis

The RLU data was analyzed by one-way ANOVA followed by Dunnett’s test for multiple comparisons of mean KIR-CD3ζ^+^ JNL cell responses to each of the Mamu-AG^+^ 721.221 cells with mean responses to the parental 721.221 cells as a negative control. For analysis of responses to the Mamu-AG2*02:01 variants, mean RLU values in response to 721.221 cells expressing each variant were compared with mean RLU values in response to 721.221 cells expressing wild-type Mamu-AG2*02:01 using an unpaired *t* test. Statistical analyses were performed using GraphPad Prism for Mac OS version 9.2.0.

## Results

### Allotypic Variation in KIR3DL05 Responses to Mamu-AG Ligands

**Figure 1:**
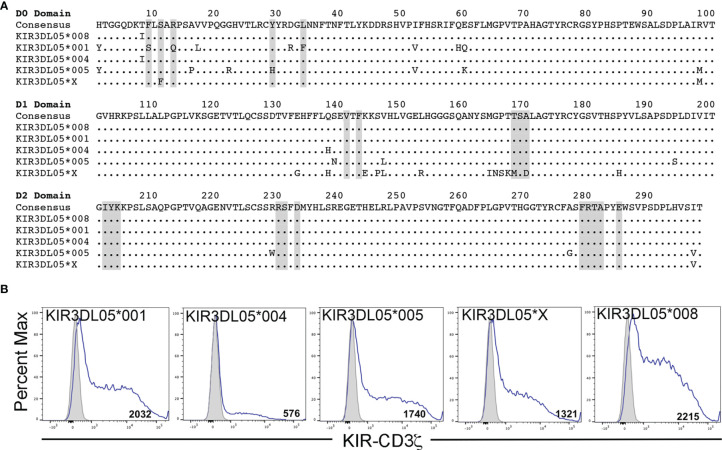
KIR3DL05 allotypes and surface expression of their KIR-CD3ζ chimeras on JNL cells. **(A)** Predicted amino acid sequence alignment for the D0, D1 and D2 domains of KIR3DL05*001, *004, *005, *X and *008. Regions shaded in gray indicate predicted MHC class I contact sites (41). Positions of amino acid identity are indicated by periods and amino acid differences are identified by their single-letter code. **(B)** KIR-CD3ζ+ JNL cells and parental JNL cells were stained with a PE-conjugated anti-Flag antibody and Near-IR LIVE/DEAD stain. After gating to exclude dead cells, the fluorescence intensity of KIR-CD3ζ (Flag-tag) staining on KIR-CD3ζ-transduced JNL cells (open) was compared to background staining on parental JNL cells (shaded). Flow cytometry data was analyzed using FlowJo 10.6 for Mac OSX. Values indicate geometric mean fluorescence intensity of staining.

**Figure 2:**
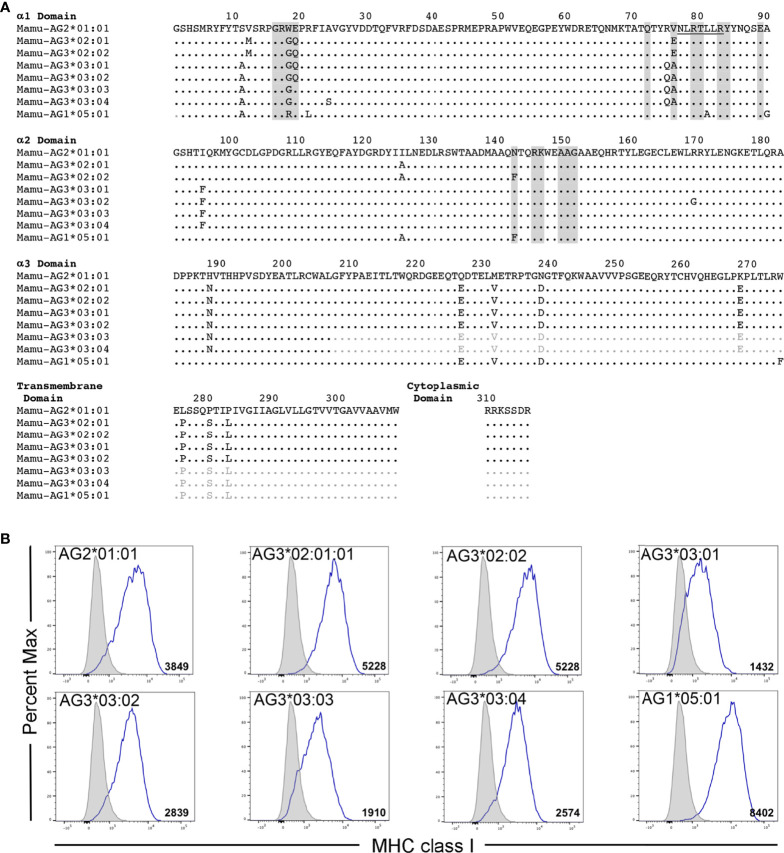
Mamu-AG allotypes and their surface expression on 721.221 cells. **(A)** An alignment showing the predicted amino acid sequences for Mamu-AG2*01:01, -AG3*02:01, -AG3*02:02, -AG3*03:01, -AG3*03:02, -AG3*03:03, -AG3*03:04, and -AG1*05:01. Regions shaded in gray indicate predicted KIR contact sites (41). Residues 77-83 that correspond to a Bw4 motif are underlined. Positions of amino acid identity are indicated by periods and amino acid differences are identified by their single-letter code. Sequences in the α3-, transmembrane and cytoplasmic domains of Mamu-AG3*03:03, -AG3*03:04, and -AG1*05:01 that were inferred from the consensus sequence are indicated in light gray. **(B)** Mamu-AG+ 721.221 cells and parental 721.221 cells were stained with a PE-conjugated pan-MHC class I-specific antibody (W6/32) and Near-IR LIVE/DEAD stain. After gating to exclude dead cells, the fluorescence intensity of MHC class I staining on Mamu-AG-transduced 721.221 cells (open) was compared to background staining of their respective parental cell lines (shaded). Flow cytometry data was analyzed using FlowJo 10.6 for Mac OSX. Values indicate geometric mean fluorescence intensity of staining.

Mamu-AG2*01:01 was previously identified as a ligand for KIR3DL05*008 (39). However, Mamu-AG and KIR3DL05 are both polymorphic, raising the possibility of allelic variation in ligand recognition. To assess the impact of polymorphisms in these molecules on receptor-ligand interactions, five allotypes of KIR3DL05 were tested for the recognition of eight allotypes of Mamu-AG. KIR3DL05*001, *004, *005, *X and *008 were selected from a previous study showing differences in KIR3DL05 binding to Mamu-A1*002-peptide complexes (40) (**Figure 1**) and Mamu-AG2*02:01, -AG3*02:01, -AG3*02:02, -AG3*03:01, -AG3*03:02, -AG3*03:03, -AG*03:04 and -AG1*05:01 were selected as representative allomorphs of predicted *Mamu-AG* loci (**Figure 2A**). Jurkat cells containing an NFAT-inducible luciferase reporter gene (JNL cells) were transduced with retroviral vectors expressing chimeric KIR-CD3ζ receptors consisting of the extracellular domains of KIR3DL05 (D0, D1, D2 and stem) fused to the transmembrane and cytoplasmic domains of human CD3ζ. To verify surface expression, a Flag-tag (DYKDDDDK) was appended to the N-terminus of the D0 domain of each of the KIR-CD3ζ chimeras (**Figure 1B**). 721.221 cells, which are deficient for the expression of endogenous HLA class I molecules (42), were in turn transduced with vectors expressing individual Mamu-AG alleles (**Figure 2B**).

**Figure 3:**
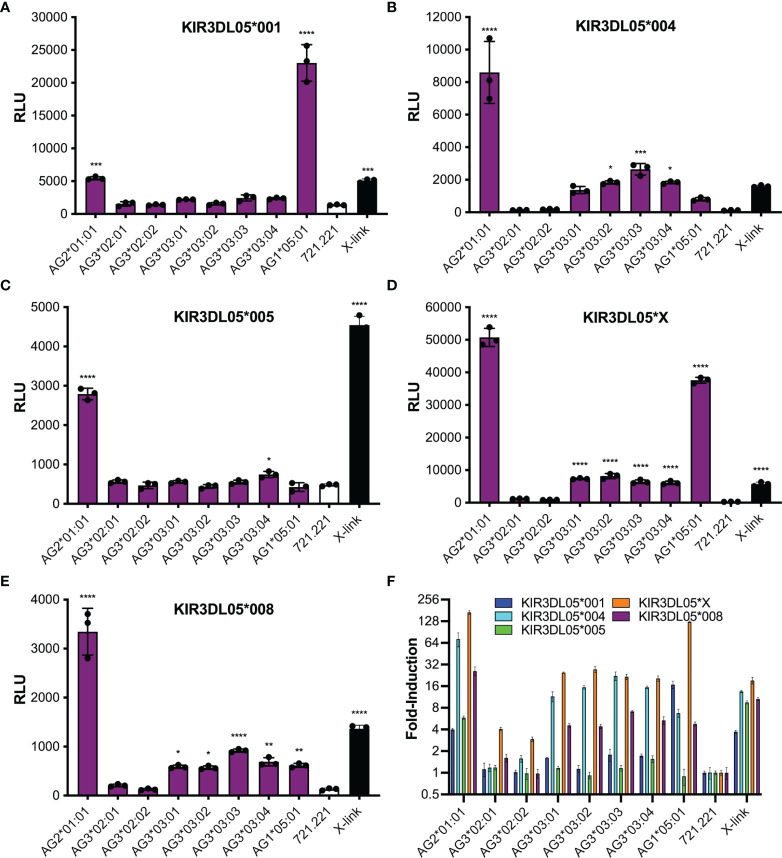
Mamu-AG recognition by five different allotypes of KIR3DL05. Jurkat NFAT luciferase (JNL) cells expressing the ectodomains of KIR3DL05*001 **(A)**, KIR3DL05*004 **(B)**, KIR3DL05*005 **(C)**, KIR3DL05*X **(D)**, or KIR3DL05*008 **(E)** fused to the transmembrane and cytoplasmic domains of CD3ζ were incubated at a 1:1 E:T ratio with 721.221 cells expressing eight different Mamu-AG allotypes. KIR-CD3ζ+ JNL cells were also incubated with parental 721.221 cells as a negative control and with a combination of anti-Flag and anti-mouse IgG antibodies as a positive control (X-link). Ligand recognition was detected by the upregulation of luciferase. **(A–E)** Bars indicate the mean relative light units (RLU) of luciferase activity in triplicate wells for each of the receptors plotted separately, or **(F)** the fold-induction of luciferase over background responses to parental 721.221 cells for each of the receptors plotted together. Error bars indicate SD of the mean and asterisks denote statistically significant differences (*p < 0.05, **p < 0.005, ***p < 0.0005, ****p < 0.0001, one-way ANOVA with Dunnett’s test).

Polymorphic differences were observed in KIR3DL05-mediated responses to different Mamu-AG molecules. Ligand recognition was detected by the MHC class I-dependent upregulation of luciferase by the KIR-CD3ζ+ JNL cells after an overnight incubation with each of the Mamu-AG+ 721.221 cells. Differences in luciferase induction were compared to parental 721.221 cells as a negative control and to KIR-CD3ζ+ JNL cells incubated with anti-Flag and anti-mouse IgG antibodies to cross-link receptors as a positive control for signaling. All five KIR3DL05 allotypes responded to Mamu-AG2*01:01 (**Figure 3**). Dominant responses to Mamu-AG1*05:01 were also observed for KIR3DL05*001 and KIR3DL05*X, and additional responses to Mamu-AG3*03:01, -AG3*03:02, -AG3*03:03 and -AG3*03:04 were detectable for KIR3DL05*004, KIR3DL05*X and KIR3DL05*008 (**Figure 3**). However, none of the KIR3DL05 allotypes responded to either Mamu-AG3*02:01 or -AG3*02:02 (**Figure 3**). These differences in ligand recognition did not correspond to differences in the surface expression of KIR-CD3ζ on JNL cells or Mamu-AG on 721.221 cells, but instead reflected polymorphisms in KIR3DL05 and Mamu-AG.

### Mamu-AG Recognition by an Activating KIR

**Figure 4:**
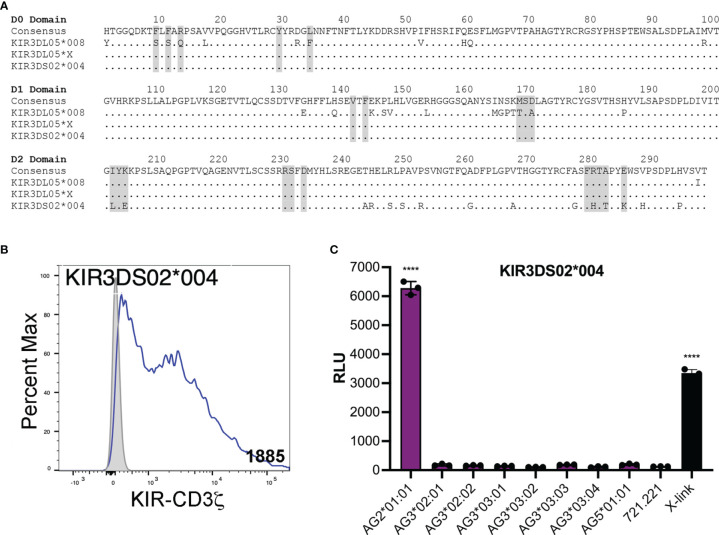
KIR3DS02 recognition of Mamu-AG. **(A)** Predicted amino acid sequence alignment for the D0-D2 domains of KIR3DL05*008, KIR3DL05*X and KIR3DS02*004. Regions shaded in gray indicate predicted MHC class I contact sites (41). Positions of amino acid identity are indicated by periods and amino acid differences are identified by their single-letter code. **(B)** KIR3DS02*004-CD3ζ+ JNL cells and parental JNL cells were stained with a PE-conjugated anti-Flag antibody and Near-IR LIVE/DEAD stain. After gating to exclude dead cells, the fluorescence intensity of KIR-CD3ζ (Flag-tag) staining on KIR-CD3ζ-transduced JNL cells (open) was compared to background staining on parental JNL cells (shaded). Geometric MFI values indicated. Flow cytometry data was analyzed using FlowJo 10.6 for Mac OSX. **(C)** KIR3DS02*004-CD3ζ+ JNL cells were incubated with 721.221 cells expressing eight different Mamu-AG allotypes at a 1:1 E:T ratio. KIR-CD3ζ+ JNL cells were also incubated with parental 721.221 cells as a negative control and with a combination of anti-Flag and anti-mouse IgG antibodies as a positive control (X-link). Ligand recognition was detected by the upregulation of luciferase. Bars indicate the mean relative light units (RLU) of luciferase activity in triplicate wells. Error bars indicate SD of the mean and asterisks denote statistically significant differences (****p < 0.0001, one-way ANOVA with Dunnett’s test).

KIR3DL05*X is the product of a recombination event that resulted in a hybrid receptor with D0 and D1 domains corresponding to KIR3DS02 and a D2 domain typical of KIR3DL05. We therefore tested KIR3DS02*004, which shares identical D0 and D1 domains with KIR3DL05*X, to determine if this activating KIR could also recognize Mamu-AG (**Figure 4A**). JNL cells expressing a KIR3DS02*004-CD3ζ fusion responded to 721.221 cells expressing Mamu-AG2*01:01, but not to cells expressing other Mamu-AG alleles (**Figures 4B, C**). These results identify Mamu-AG2*01:01 as a ligand for KIR3DS02*004 and suggest that the more restricted pattern of responses to Mamu-AG compared to KIR3DL05*X reflects differences in the D2 domain.

### Mamu-AG Determinants of Recognition by KIR3DL05

**Figure 5:**
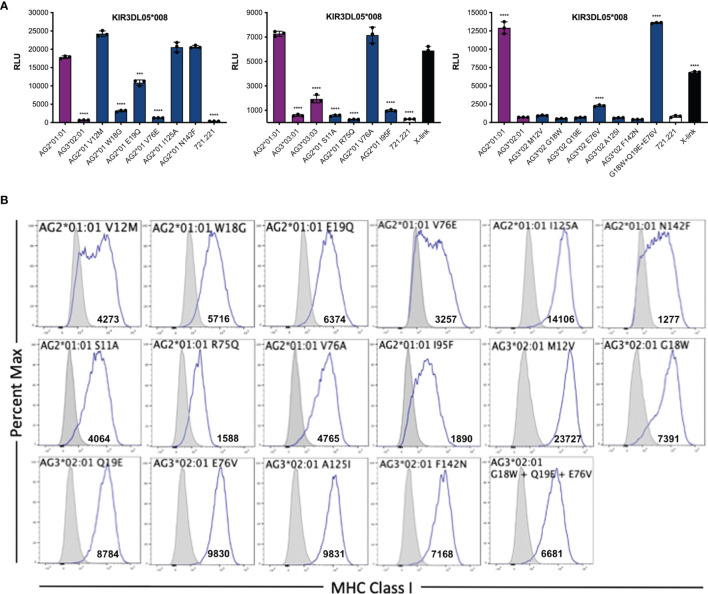
Identification of residues in the α1- and α2-domains of Mamu-AG that contribute to recognition by KIR3DL05. **(A)** 721.221 cells expressing Mamu-AG2*01:01 variants with single amino acid substitutions at positions that differ from Mamu-AG3*02:01 or -AG3*03:01 and Mamu-AG3*02:01 variants with substitutions at positions that differ from Mamu-AG2*01:01 were co-incubated with of KIR3DL05*008-CD3ζ+ JNL cells overnight at a 1:1 E:T ratio. KIR-CD3ζ+ JNL cells were also incubated with parental 721.221 cells as a negative control and with anti-Flag and anti-mouse IgG antibodies as positive control (X-link). Bars represent the mean luciferase activity (RLU) from triplicate wells. Error bars indicate SD of the mean and asterisks denote statistically significant differences (***p < 0.0005, ****p < 0.0001, one-way ANOVA with Dunnett’s test). **(B)** Mamu-AG+ 721.221 cells and parental 721.221 cells were stained with a PE-conjugated pan-MHC class I-specific antibody (W6/32) and Near-IR LIVE/DEAD stain. After excluding dead cells, the fluorescence intensity of MHC class I staining on the surface of Mamu-AG-transduced 721.221 cells (open) was compared to background staining on parental 721.221 cells (shaded). Flow cytometry data was analyzed using FlowJo 10.6 for Mac OSX. Values indicate geometric mean fluorescence intensity of staining.

To define Mamu-AG polymorphisms that account for differences in KIR3DL05 recognition, amino acid substitutions were introduced into Mamu-AG2*01:01 at positions that differ from Mamu-AG3*02:01 and -AG3*03:01. 721.221 cells expressing these variants were then tested for recognition by KIR3DL05-CD3ζ+ JNL cells (**Figure 5**). The replacement of any one of three amino acids in the α1-domain of Mamu-AG2*01:01 with the corresponding residues of Mamu-AG3*02:01 (W18G, E19Q or V76E) partially or completely impaired KIR3DL05 recognition (**Figure 5A**). Likewise, individually exchanging three amino acids of Mamu-AG2*01:01 with the corresponding residues of Mamu-AG3*03:01 (S11A, R75Q or I95F) significantly impaired interactions with KIR3DL05 (**Figure 5A**). Conversely, a combination of three amino acid changes in the α1-domain of Mamu-AG3*02:01 (G18W, Q19E and E76V) was sufficient to restore interactions with KIR3DL05 to a similar level as Mamu-AG2*01:01 (**Figure 5A**). Thus, similar to the KIR recognition of classical MHC class I ligands, the specificity of KIR3DL05 is primarily determined by polymorphisms in the α1-domain of Mamu-AG.

**Figure 6:**
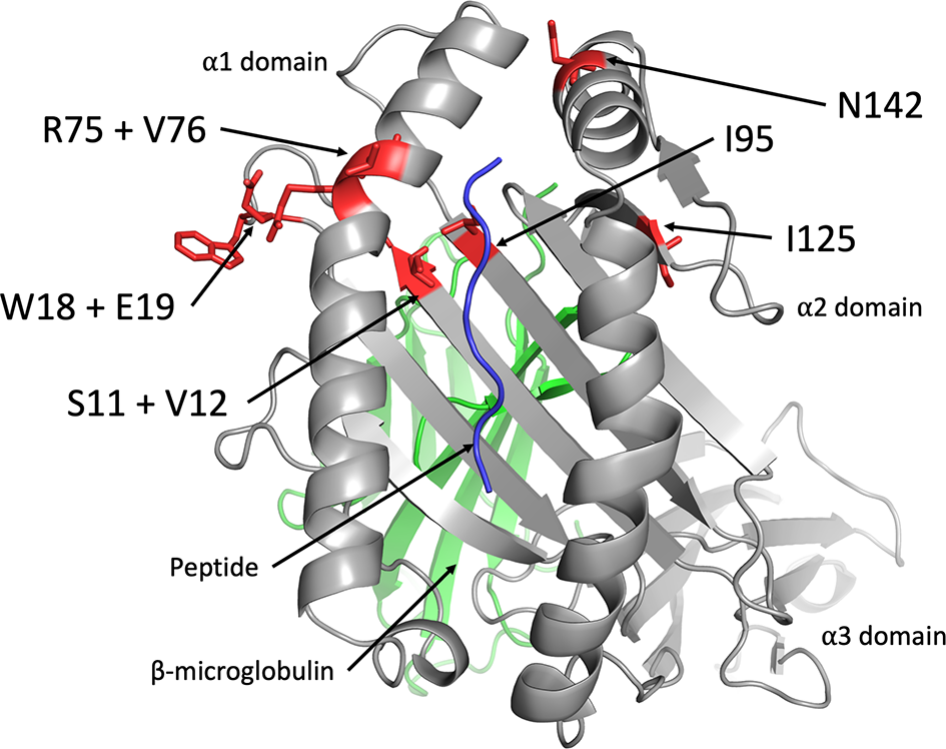
Location of amino acid polymorphisms in Mamu-AG that affect recognition by KIR3DL05. The side chains of Mamu-AG2*01:01 residues that affect recognition by KIR3DL05*008 (red) were modeled on a three-dimensional crystal structure of Mamu-A1*002 (grey) in complex with peptide (blue) and β2-microglobulin (green) (PDB 3jtt) using PyMol software.

The amino acid residues of Mamu-AG2*01:01 that participate in interactions with KIR3DL05 were modeled by *in silico* replacement of the corresponding residues in a three-dimensional structure of Mamu-A1*002. Mamu-A1*002 was selected for modeling due to its general sequence similarity with Mamu-AG and because it is also a ligand for KIR3DL05 (43). All of the polymorphic residues that affect KIR3DL05 recognition are in close proximity to the Bw4 motif (residues 77-83) at the C-terminal end of the α1-domain (**Figure 6**). Residues 75 and 76, for which the R75Q and V76E polymorphisms had the greatest impact on KIR3DL05 responses, are part of the α-helical region directly adjacent to the Bw4 motif (**Figure 6**). Residues 18 and 19 are located in an underlying loop that was previously identified as a KIR contact site based on the crystal structure of human KIR3DL1 in complex with HLA-B*5701 (**Figure 6**) (41). The side chains of these residues are surface exposed in a region where the W18G and E19Q polymorphisms would be expected to contribute to a patch of sequence diversity together with polymorphisms at positions 75 and 76. In contrast, residues 11 and 95 are located in the floor of the peptide binding pocket and are unlikely to be surface exposed. The partial effects of the S11A and I95F on KIR3DL05 responses may therefore reflect differences in peptide binding and/or conformational changes to the peptide-MHC class I complex. Overall, these results are consistent with the structure of KIR3DL1 in complex with HLA-B*5701 showing extensive contacts between the D0 and D1 domains of the KIR and the α1-domain of the MHC class I molecule (41). These findings also illustrate that while polymorphisms in residues 77-83 may determine broad patterns of KIR recognition (e.g. Bw4 specificity), polymorphisms in adjoining regions can contribute to variation in responses to closely related MHC class I allotypes.

## Discussion

Definition of the MHC class I ligands of rhesus macaque KIRs is foundational for studying NK cell responses in this species as an animal model for infectious diseases, transplantation and reproductive biology. In a previous survey of KIR-MHC class I interactions in the rhesus macaque, Mamu-AG2*01:01 was identified as a ligand for KIR3DL05*008 (39). Although Mamu-AG does not have a direct human ortholog, it shares similar molecular features and tissue distribution as HLA-G (19, 20, 23). However, Mamu-AG is considerably more polymorphic than HLA-G owing to allelic variation at multiple loci (44). KIR3DL05 is also polymorphic with at least 33 different *KIR3DL05* alleles identified so far (45). To gain a better understanding of the determinants of KIR3DL05 specificity for Mamu-AG, we investigated the impact of Mamu-AG polymorphisms on this receptor-ligand interaction.

Our findings reveal three general patterns of Mamu-AG recognition among the KIR3DL05 allotypes selected for this study. The most common pattern exhibited by KIR3DL05*004, *005 and *008 includes dominant responses to Mamu-AG2*01:01 and lower, more variable responses to Mamu-AG3*03:01, -AG3*03:02, -AG3*03:03 and -AG3*03:04. The nearly identical responses of KIR3DL05*004 and *008 reflect the overall similarity of their extracellular domains, which only differ by a single amino acid in the D1 domain (Q139H). KIR3DL05*005 differs from each of these KIRs by seven residues in D0 domain and two in the D1 domain. However, only one of these polymorphisms (Y29H) is located in a predicted MHC class I contact site. In contrast to the other allotypes, KIR3DL05*001 responded strongly to Mamu-AG1*05:01, weakly to Mamu-AG2*01:01, and not at all to Mamu-AG3. Since KIR3DL05*001 and *008 share identical D1 and D2 domains, but differ by nine amino acids in the D0 domain, including three residues in predicted MHC class I contact sites, this shift in Mamu-AG recognition is a function of polymorphisms in the D0 domain. KIR3DL05*X exhibited a more complex pattern that included dominant responses to both Mamu-AG2*01:01 and -AG1*05:01, and detectable responses to Mamu-AG3*03:01, -AG3*03:02, -AG3*03:03 and -AG3*03:04. Whereas KIR3DL05*X differs from KIR3DL05*008 by only two amino acids in the D0 domain, there are thirteen differences in the D1 domain. The more extensive differences in D1 reflect the hybrid origins of this KIR, which is comprised of the leader peptide, D0 and D1 domains of KIR3DS02 (exons 1-4) and the D2, stem, transmembrane and cytoplasmic domains of KIR3DL05 (exons 5-9) (40). Overall, these observations reveal differences in KIR3DL05 recognition of Mamu-AG molecules that are determined by polymorphisms in their D0 and D1 domains.

We also tested an allotype of KIR3DS02 with identical D0 and D1 domains to KIR3DL05*X. KIR3DS02*004 recognized Mamu-AG2*01:01, but did not respond to other Mamu-AG allotypes. These results identify Mamu-AG2*01:01 as a ligand for this activating KIR and indicate that differences in the D2 domains of KIR3DS02*004 and KIR3DL05*X account for differences in their responses to other Mamu-AG molecules.

An analysis of single amino acid variants identified Mamu-AG polymorphisms that affect KIR3DL05 recognition. These include differences in the α-helical region of the α1 domain (R75Q & V76E), an N-terminal loop of the α1 domain (W18G & E19Q), and the β-sheet that forms the floor of the peptide binding pocket (S11A & I95F). Residues 18, 19, 75 and 76 are located on the same face of the α1 domain and coincide with predicted KIR contact sites (41). Thus, the effects of polymorphisms at these positions may be attributed to interactions with KIR3DL05. On the other hand, residues 11 and 95 are not predicted to contact the KIR, but may alter the conformation of the bound peptide or adjacent residues in the α1 domain in a manner that indirectly affects interactions with KIR3DL05. These observations illustrate how a few amino acid differences in surfaces of the α1 domain can result in differential recognition of closely related MHC class I molecules and are consistent with the co-evolution of the D0 and D1 domains of KIRs in concert with the α1 domain of MHC class I ligands.

In humans, HLA-G is expressed by fetal trophoblasts that invade the uterine endometrium during early stages of pregnancy and on extravillous trophoblasts that remodel spiral arteries of the developing placenta (46). HLA-G is a ligand for KIR2DL4 (47, 48), which is an unusual 2 domain KIR with both activating and inhibitory features. KIR2DL4 has a positively charged arginine residue in the transmembrane domain for pairing with FcεRIγ to transduce activating signals and a long cytoplasmic tail with an immunotyrosine-based inhibitory motif (ITIM) (49). This receptor interacts with a soluble isoform of HLA-G in endosomes to stimulate the release of pro-inflammatory and pro-angiogenic factors by decidual NK cells that promote placental vascularization (50–52). Although the presence of HLA-E on extravillous trophoblasts is thought to play a dominant role in maintaining fetal tolerance *via* inhibitory interactions with CD94/NKG2A on decidual NK cells, HLA-G may facilitate tolerance through additional interactions with inhibitory receptors and by serving as a source of leader peptides bound by HLA-E (52, 53).

The ortholog of *HLA-*G (*Mamu-G*) in rhesus macaques is a pseudogene (17). However, macaques express another nonclassical MHC class I molecule with similar tissue distribution (19). Like HLA-G, Mamu-AG is expressed on fetal cytotrophoblasts that invade the endometrium and on syncytiotrophoblasts of the placental chorionic villi (23). While it has been difficult to experimentally verify the function of HLA-G in humans, passive transfer of a Mamu-AG-specific monoclonal antibody to pregnant macaques was shown to disrupt leukocyte responses to implantation, vascularization, and placental development (38). Thus, Mamu-AG appears to serve a similar function as HLA-G.

The identification of Mamu-AG as a ligand for KIR3DL05 and KIR3DS02 reveals that certain macaque KIRs are capable of mediating functional interactions with this nonclassical molecule. While macaques express a recognizable ortholog of KIR2DL4 (KIR2DL04) (7, 54), there is no reason to believe that this receptor interacts with Mamu-AG, which bears no homology to HLA-G. Nevertheless, it is likely that there are other receptors for Mamu-AG that are yet to be identified. These may include additional KIRs or other types of receptors that react more broadly with MHC class I molecules, such as the Ig-like transcripts 2 and 4 (ILT-2 and ILT-4) that are known to serve as receptors for HLA-G in humans (55).

A complex and precarious balance of NK cell activating and inhibitory receptor interactions is essential for normal placental development, fetal tolerance, and immune surveillance of the maternal-fetal interface. Precisely how KIR3DL05 and KIR3DS02 interactions with Mamu-AG contribute to this balance in macaques is presently unclear. The ligation of activating receptors on decidual NK cells may stimulate the release of pro-inflammatory and pro-angiogenic factors that promote placental vascularization and increase blood supply to the developing fetus, similar to the interactions of KIR2DL4 with soluble HLA-G or lineage III KIRs (KIR2Ds) with HLA-C in humans (52, 56). In the absence of HLA-G or HLA-C, it is conceivable that macaques depend on Mamu-AG recognition by activating KIRs such as KIR3DS02 to stimulate placental vascularization. Mamu-AG may also facilitate fetal tolerance through interactions with inhibitory KIRs such as KIR3DL05 or by providing leader peptides that stabilize Mamu-E on the surface of trophoblasts for inhibitory signaling through CD94/NKG2A (57).

Recent studies have demonstrated a role for decidual NK cells in protecting the maternal-fetal interface. Extravillous trophoblasts are susceptible to a number of pathogens that are transmitted *in utero*, including Zika virus (ZiKV), cytomegalovirus (CMV), and *Toxoplasma gondii* (58–63). In the case of ZiKV, infection of extravillous trophoblasts causes ER stress that results in the downregulation of HLA-C and -G, which renders these cells susceptible to killing by decidual NK cells (60). Similar antimicrobial functions of decidual NK cells have been reported for CMV and *Listeria monocytogenes* (64–66). Thus, disruption or alteration of Mamu-AG expression on macaque trophoblasts may trigger decidual NK cell responses through inhibitory or activating KIRs. KIR3DL05 and KIR3DS02 may thereby contribute to pregnancy success by protecting the maternal-fetal interface from invading microorganisms. Furthermore, polymorphic differences in these KIRs and Mamu-AG could account for individual variation in resistance to placental transmission of viral and bacterial pathogens.

The rhesus macaque has become an increasingly valuable animal model for studying viral pathogens such as CMV and Zika virus that are frequently transmitted to the developing fetus during pregnancy (67–70). Hence, the molecular interactions between macaque NK cell receptors and their MHC class I ligands that occur at the maternal-fetal interface may be especially important for understanding viral transmission and pathogenesis in this context. The present study defines many of the Mamu-AG ligands recognized by diverse allotypes of KIR3DL05 and identifies KIR3DS02*004 as an activating receptor for Mamu-AG2*01:01. These findings afford greater insight into NK cell recognition of a nonclassical molecule implicated in pregnancy and provide a broader foundation for investigating NK cell biology in the rhesus macaque as a nonhuman primate model for infectious diseases and reproductive biology.

## Data Availability Statement

The GenBank accession numbers for the sequences presented in this study are provided in the *Materials and Methods*. All of the data presented in this study are included in the article. Further inquires can be directed to the corresponding author.

## Author Contributions

RN, JA, WS, MW, and PB performed the experiments and analyzed the data. KS and SJ designed the experiments. DE conceived the project and wrote the manuscript. All of the authors contributed to the manuscript and approved the submitted version.

## Funding

This work was supported by Public Health Service Grants AI095098, AI161816, AI121135, AI148379 and AI098485 (to DE) and OD011106 (to the WNPRC).

## Conflict of Interest

The authors declare that the research was conducted in the absence of any commercial or financial relationships that could be construed as a potential conflict of interest.

## Publisher’s Note

All claims expressed in this article are solely those of the authors and do not necessarily represent those of their affiliated organizations, or those of the publisher, the editors and the reviewers. Any product that may be evaluated in this article, or claim that may be made by its manufacturer, is not guaranteed or endorsed by the publisher.
